# Supplementary figures and images for: No Association between ABCB1 G2677T/A or C3435T Polymorphisms and Survival of Breast Cancer Patients—A 10-Year Follow-Up Study in the Polish Population
Source: Genes (Basel). 2022 Apr 21;13(5):729. doi: 10.3390/genes13050729 (PMC9141033; doi:10.3390/genes13050729)

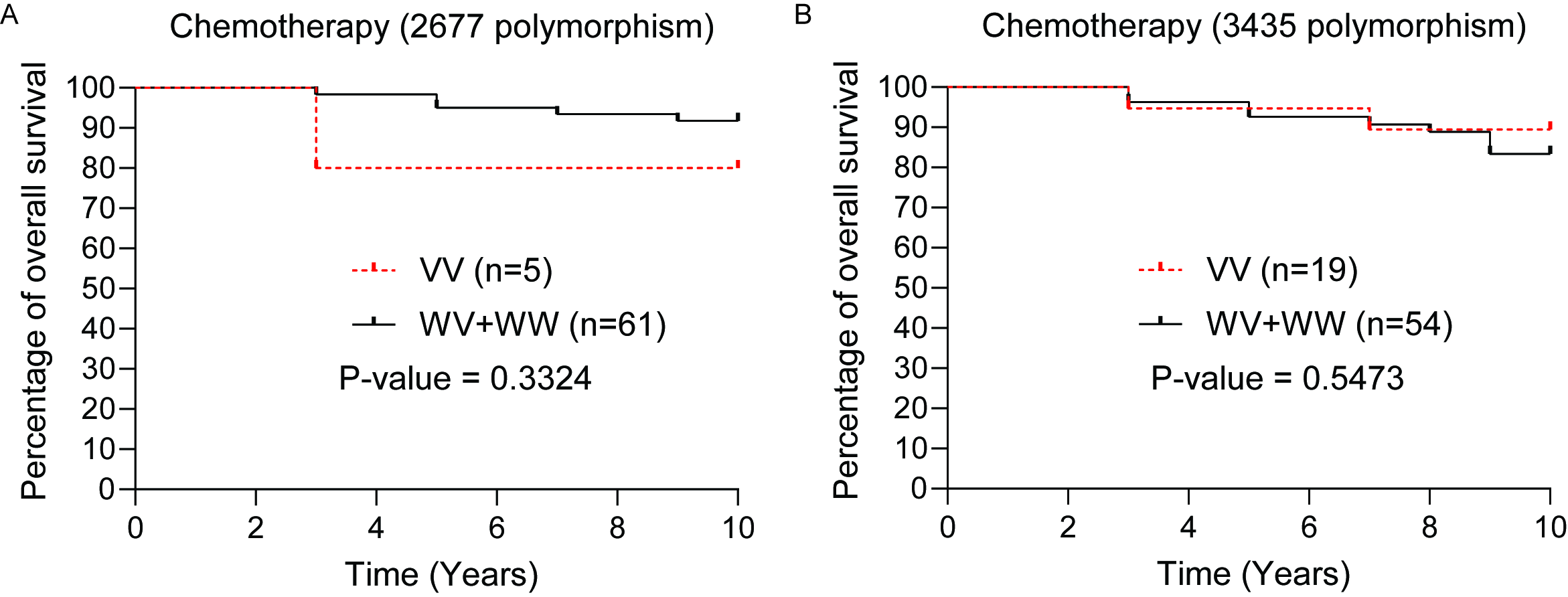

Supplement: Supplementary file 1 [file genes-13-00729-s001.zip › genes-1645806/FigS1.tif]
